# A Herbal Formula HT048, *Citrus unshiu* and *Crataegus pinnatifida*, Prevents Obesity by Inhibiting Adipogenesis and Lipogenesis in 3T3-L1 Preadipocytes and HFD-Induced Obese Rats

**DOI:** 10.3390/molecules20069656

**Published:** 2015-05-26

**Authors:** Yoon Hee Lee, Young-Sik Kim, Mikyung Song, Minsu Lee, Juyeon Park, Hocheol Kim

**Affiliations:** 1 Korea Institute of Science and Technology for Eastern Medicine (KISTEM), NeuMed Inc., Seoul 130-701, Korea; E-Mails: eiyoon88@naver.com (Y.H.L.); saeim@naver.com (J.P.); 2 Department of Herbal Pharmacology, College of Korean Medicine, Kyung Hee University, Seoul 130-701, Korea; E-Mails: yjbsik@naver.com (Y.-S.K.); ssong8230@gmail.com (M.S.); lmins357@hanmail.net (M.L.)

**Keywords:** anti-obesity, herbal combination, HT048, *Citrus unshiu*, *Crataegus pinnatifida*

## Abstract

HT048 is a combination composed of *Crataegus pinnatifida* leaf and *Citrus unshiu* peel extracts. This study aimed to investigate potential anti-obesity effect of the combination. The 3T3-L1 adipocytes were treated with different doses of HT048 and triglyceride accumulation, glycerol release and adipogenesis-related genes were analyzed. For *in vivo* study, male Sprague Dawley rats were divided according to experimental diets: the chow diet group, the high-fat diet (HFD) group, the HFD supplemented with orlistat group, the HFD supplemented with HT048 group (0.2% or 0.4%) for 12 weeks. We measured the body weight, serum lipid levels and the expression of genes involved lipid metabolism. HT048 treatment dose-dependently suppressed adipocyte differentiation and stimulated glycerol release. The expressions of PPARγ and C/EBPα mRNA were decreased by HT048 treatment in adipocytes. HT048 supplementation significantly reduced the body and fat weights *in vivo*. Serum lipid levels were significantly lower in the HT048 supplemented groups than those of the HFD group. Expression of the hepatic lipogenesis-related genes were decreased and expression of the β-oxidation-related genes were increased in rats fed HT048 compared to that of animals fed HFD. These results suggest that HT048 has a potential benefit in preventing obesity through the inhibition of lipogenesis and adipogenesis.

## 1. Introduction

In recent years, obesity is the most common metabolic disease emerging as a global problem, not only in developed countries but also in developing countries. Obesity is associated with various health problems, such as diabetes, hypertension, cardiovascular disease (CVD), and certain forms of cancer [1]. It is characterized by an increase in excessive accumulation of fat in a liver and elevated lipid concentrations in a blood, which can result from an imbalance between energy intake and expenditure [2,3]. An elevation of serum triglyceride (TG), total cholesterol (TC) and low-density lipoprotein cholesterol (LDL-C) levels induces the elevated blood lipid concentrations [4]. Increased blood free fatty acid (FFA) levels induces insulin resistance and enhances delivery of FFA to a liver, and up-regulate hepatic lipogenic gene expression, including sterol regulatory element-binding protein 1c (SREBP1c) and fatty acid synthase (FAS) [5].

The process of adipose tissue accumulation is caused by an increased adipogenesis accompanied by adipocyte differentiation, which generates mature adipocyte from preadipocytes. Expression of a cascade of transcription factors involved in adipocyte differentiation includes peroxisome proliferator-activated receptor-gamma (PPARγ) and CCAAT/enhancer binding protein-alpha (C/EBPα) [6]. This transcription factor regulates the lipid homeostasis by modulating the expression of target genes, including FAS, activating protein 2 (aP2) and lipoprotein lipase (LPL), associated with fat accumulation. Inhibition of adipocyte differentiation is considered to be important anti-obesity mechanism.

Up to now, many kinds of medicines has been used to prevent and treat the obesity. Clinically available anti-obesity agents including sibutramine, which is a serotonin reuptake inhibitors (SRIs), and orlistat, which is a lipase inhibitor, has been reported to come with side-effects, including gastro-intestinal discomforts, insomnia, headaches, flatulence, and diarrhea [7,8]. Therefore, it is necessary to identify the natural products with minimum side effects. HT048 is a combined formula for the treatment and management of obesity, composed of Crataegus pinnatifida leaf and Citrus unshiu peel. Hawthorn is a traditional medicine used to treat hyperlipidemia, chronic heart failure and various digestive ailments [9,10]. Recent studies showed that Hawthorn has an ability to reduce the plasma levels of triglycerides and low-density lipoprotein and improve obesity [10]. Citrus flavonoids are the polyphenolic compounds with powerful bioavailability in the treatment of dyslipidemia, insulin resistance and obesity [11,12].

In our previous study, HT048, combined *C. pinnatifida* fruit and *C. unshiu* peel, reduced body and epididymal fat weights and serum lipid levels in HFD-induced obese rat [13]. However, synergistic effect of HT048 and anti-obesity mechanism of the combination are not clearly elucidated yet. In the present study, we investigate whether HT048 synergistically ameliorates obesity by inhibiting adipogenesis and lipogenesis in 3T3-L1 preadipocytes and SD rat fed high fat diet (HFD), a widely used animal model for obesity [14]. To elucidate potential underlying mechanisms of HT048, we examined lipid droplet formation, TG accumulation and the expression of adipogenesis-related genes in 3T3-L1 adipocytes. Next, we evaluated body weight, white adipose tissue (WAT) weight and serum lipid levels, with the expression of genes involved adipogenesis and lipogenesis in liver and WAT of HFD-induced obese rats.

## 2. Results and Discussion

### 2.1. HPLC Analysis of HT048 

**Figure 1:**
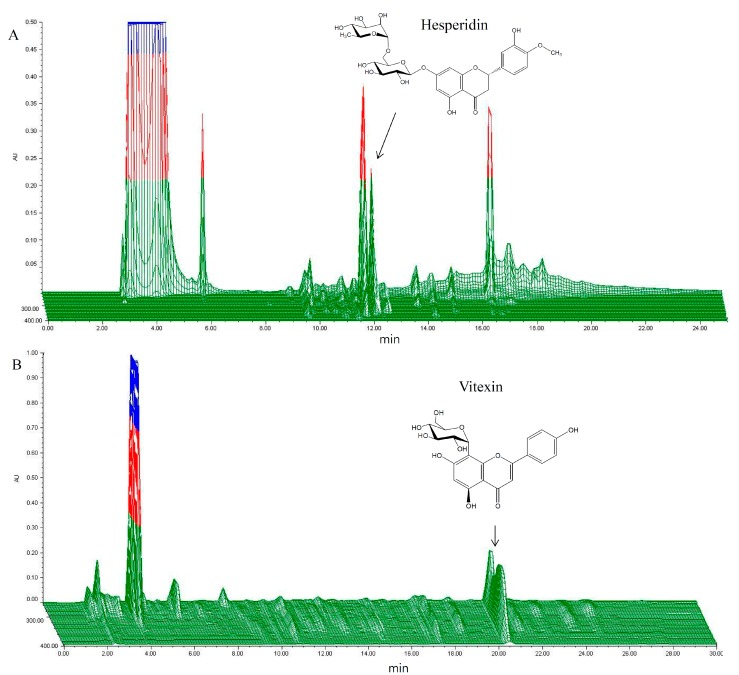
3-D HPLC chromatogram of HT048, a blend of two herbal extracts (**A**) *C. unshiu* peel of hesperidin; (**B**) *C. pinnatifida* leaf of vitexin).

The quality of HT048 was standardized using two representative components: 4.28 ± 0.11 mg/g of vitexin for *C. pinnatifida* and 4.74 ± 0.15 mg/g of hesperidin for *C. unshiu*. A 3-D HPLC chromatogram and the structures of the constituent compounds are shown in Figure 1.

### 2.2. Effect of HT048 on Adipocyte Differentiation in 3T3-L1 Adipocytes

**Figure 2:**
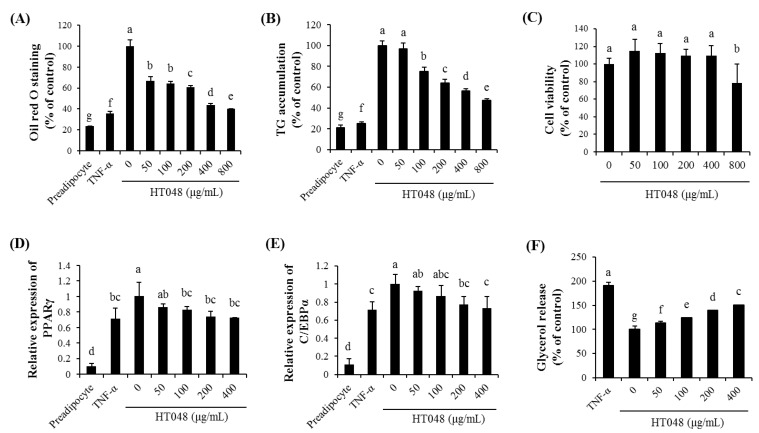
Effect of HT048 on cell viability and adipocyte differentiation of 3T3-L1 adipocyte. The 3T3-L1 adipocytes were incubated with or without HT048 of various concentrations for 24 h. Differentiated adipocytes were treated with or without HT048 of various concentrations. Oil Red O staining (**A**) and TG accumulation (**B**) of the 3T3-L1 cells were determeined. Growth rate was assessed by MTT assay (**C**). The expressions of the PPARγ (**D**) and C/EBPα (**E**) were measured by qPCR. Glycerol release assay (**F**) of the 3T3-L1 cells were determined. Values are presented as the mean ± S.D. ^abcde^^fg^ Means not sharing common letters are significantly different among the groups at *p* < 0.05.

To evaluate the effect of HT048 on adipocyte differentiation in vitro, post-confluent 3T3-L1 preadipocytes were maintained in adipocyte differentiation induction media and exposed to various doses of HT048 (0, 50, 100, 200, 400 and 800 μg/mL). Then lipid droplet and TG content were observed (Figure 2A,B). Various concentrations of HT048 (100, 200, 400 and 800 μg/mL) inhibited the lipid droplet formation significantly in a dose-dependent manner. TG accumulation were reduced in the adipocytes treated with 100, 200, 400 and 800 μg/mL of HT048 compared to differentiated untreated adipocytes in a dose-dependent manner. To exclude a possibility of the decreases in lipid droplet and TG accumulations resulting from cytotoxicity, we examined intracellular toxicity by MTT assay (Figure 2C). The treatment with HT048 at concentration of 50 to 400 μg/mL did not significantly affect the cell viability of 3T3-L1 adipocytes.

### 2.3. Effect of HT048 on the Expression of the Adipogenic Transcription Genes in 3T3-L1 Adipocytes

To determine the effect of HT048 on the expression of adipocyte differentiation related genes, we measured the expression of PPARγ and C/EBPα mRNA. As shown in Figure 2D,E, the expression of PPARγ mRNA was decreased by 100, 200 and 400 µg/mL of HT048 and the expression of C/EBPα mRNA was decreased by 200 and 400 µg/mL of HT048 in 3T3-L1 mature adipocytes.

### 2.4. Effect of HT048 on Lipolysis in 3T3-L1 Adipocytes

To examine whether HT048 is effective on lipolysis in 3T3-L1 adipocytes, various concentrations of HT048 (0, 50, 100, 200 and 400 µg/mL) were evaluated (Figure 2F). As compared with mature adipocytes, HT048 effectively stimulated glycerol release in 3T3-L1 cells in a dose-dependent manner. HT048 at concentration of 400 µg/mL showed the highest glycerol release content of adipocytes.

### 2.5. Effect of HT048 on Body and Organ Weights and Fecal Lipid Content in HFD-Induced Obese Rats

**Figure 3:**
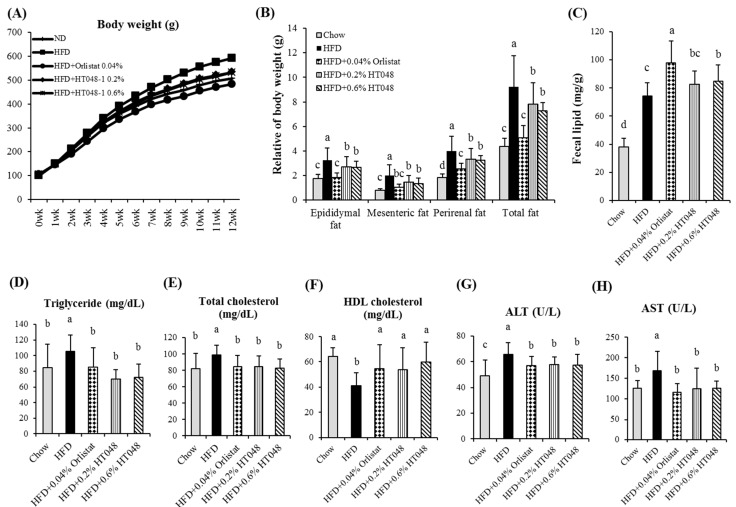
Effect of HT048 on body weight (**A**), WAT (**B**) weights, fecal lipid content (**C**) and serum parameters (**D**–**H**) in HFD-induced obese rats. Values are presented as the mean ± S.D. (*n* = 12). ^abc^ Means not sharing common letters are significantly different among the groups at *p* < 0.05.

At the end of the experiment, the body weights of the HFD group were greater than the control group by 16.8%. There were significant difference in the body weights between the HT048 supplementation groups and the HFD group by 8 weeks after initiating the experiment. The final body weights were significantly lowered by supplemented with 0.2% and 0.6% HT048 (9.9% and 10.2%, respectively) compared to the HFD control rats. In addition, orlistat supplementation significantly reduced the body weight of animals fed HFD (18.3%) (Table 1). HFD significantly increased the relative weight of visceral white adipose tissue (WAT) compared to the chow diet (10.5%). Supplementation of 0.2% and 0.6% HT048 markedly reduced the visceral fat mass (15.4% and 21.3%) including epididymal (16.7% and 18.5%), perirenal (16.6% and 18.6%), and mesenteric (26.6% and 32.2%) WAT compared to the HFD group (Figure 3B). There was no significant difference in food intakes among the HFD-fed groups (Table 1). The fecal lipid level is shown in Figure 3C. The fecal total lipid in the 0.2% HT048, 0.6% HT048 and orlistat supplementation groups were higher than HFD group (11.37%, 14.08% and 32.86%).

**Table 1:** Effect of HT048 on body weight and food intake in rat fed HFD for 12 weeks.

	Chow	HFD	HFD0.04% Orlistat	HFD0.2% HT048	HFD0.6% HT048
Food intake (g/day)	22.07 ± 0.68 ^a^	18.90 ± 0.21 ^b^	20.80 ± 1.97 ^a^	18.18 ± 0.36 ^b^	18.17 ± 1.26 ^b^
Initial body weight (g)	91.83 ± 5.37	91.92 ± 6.52	91.67 ± 4.48	91.92 ± 6.64	91.75 ± 6.92
Final body weight (g)	506.92 ± 26.37 ^bc^	592.42 ± 61.47 ^a^	483.83 ± 42.45 ^c^	533.75 ± 63.21 ^b^	532.00 ± 47.21 ^b^

Values are presented as the mean ± S.D. (*n* = 12). ^abc^ Means not sharing common letters are significantly different among the groups at *p* < 0.05.

### 2.6. Effect of HT048 on Serum Measurements in HFD-Induced Obese Rats

Similar to the results for body weight and fat mass, serum TG, TC, ALT and AST levels were significantly higher and HDL-C was significantly lower in the HFD group compared to the other groups (Figure 3D–H). The supplementation of 0.2% and 0.6% HT048 significantly reduced the serum TG (33.7% and 31.9%), TC (14.4% and 16.3%), ALT (12.4% and 13%), and AST (25.9% and 25.2%) levels in HFD-fed animals compared to the HFD-control group, whereas serum HDL-C (23.6% and 31.5%) level was significantly higher in the HT048 groups compared to the HFD group.

### 2.7. Effect of HT048 on Hepatic Expression of Lipogenesis-Related Genes in HFD-Induced Obese Rats

**Figure 4:**
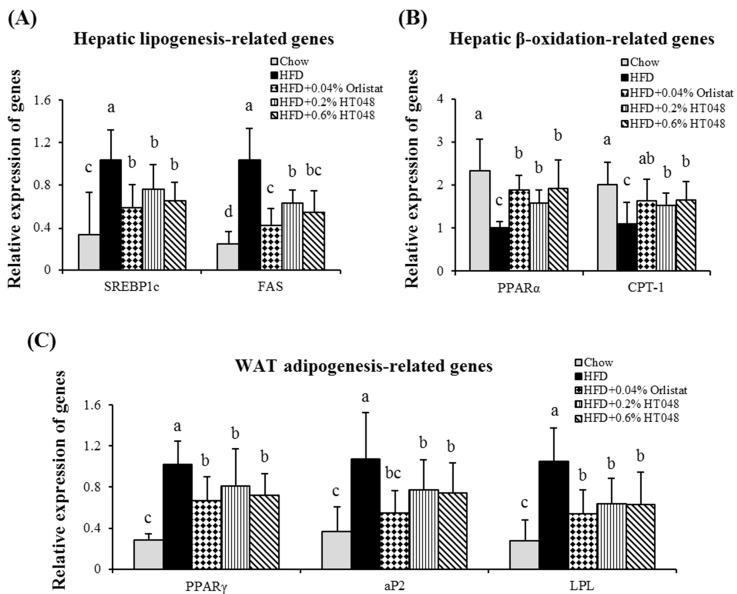
Effect of HT048 on mRNA expression of genes related to hepatic lipogenesis (**A**) and β-oxidation (**B**) and WAT adipogenesis (**C**) in HFD-induced obese rats. Values are presented as the mean ± S.D. (*n* = 12). ^abc^ Means not sharing common letters are significantly different among the groups at *p* < 0.05.

We investigated the potential mechanisms by which the HT048 might attenuate the HFD-induced activation of lipogenic genes including SREBP1c and FAS. These two lipogenic genes were significantly higher in HFD group compared to the chow diet group. HT048 supplementation reduced the expression levels of SREBP1c and FAS mRNA in the animals fed HFD (Figure 4A). Furthermore, the expressions level of β-oxidation genes including PPARα and CPT-1 were significantly decreased by HFD (Figure 4B). HT048 supply induced significant increases in the expression of PPARα and CPT-1 in rats fed HFD.

### 2.8. Effect of HT048 on Adipose Tissue Expression of Adipogenesis-Related Genes in HFD-Induced Obese Rats

To understand the effect of HT048 on anti-adipogenesis, we assessed the changes in the expression of genes involved in adipocyte differentiation and fat accumulation in WAT. As we expected, HFD group showed the highest expressions of PPARγ, aP2, and LPL mRNA and HT048 supplementation significantly suppressed the expression of these genes in the HFD-fed animals (Figure 4C).

### 2.9. Discussion

Adipogenesis is a part of adipocyte differentiation process from preadipocyte precursors into mature adipocytes with the formation and enlargement of intracellular lipid droplets [15]. This process is associated with the development of obesity. HT048 effectively inhibited the lipid droplet formation and TG accumulation in the 3T3-L1 adipocytes. The 3T3-L1 adipocytes are commonly used for investigating the molecular mechanisms of adipogenesis [16]. To examine the effects of the HT048 on the adipocyte differentiation, post-confluent 3T3-L1 preadipocytes were treated with various doses of HT048 and then lipid droplet and TG content were analyzed. HT048 significantly inhibits adipocyte differentiation in 3T3-L1 preadipocytes. According to the MTT assay results, the reduction of lipid droplet formation and TG accumulation in HT048 treated adipocyte at concentration of 50 to 400 μg/mL was not due to cellular toxicity. In addition, we observed that HT048 treatment increased the lipolysis as measured by glycerol release determination in 3T3-L1 adipocytes. Lipolysis is one of the most important catabolic process to reduce adipose mass, leads to breakdown the stored TG in adipocytes and release of FFA and glycerol [17]. This data revealed that HT048 possessed the anti-obesity effects by inhibiting the lipid formation and promoting the lipolysis in 3T3-L1 adipocytes.

Adipogenesis is controlled by a several transcription factors such as PPARγ and C/EBPs [18,19], which are mostly expressed in adipose [15]. In the middle stage of adipogenesis, PPARγ and C/EBPα synergistically active the expression of adipocyte marker genes to transform preadipocytes into mature adipocytes [20]. Our data showed that during adipogenesis, HT048 effectively reduced the expression of PPARγ and C/EBPα mRNA. Therefore, it is suggested that HT048 inhibits adipogenesis through the down-regulation of adipogenic transcriptional factors in adipocyte differentiation.

We used HFD-induced obesity rat model to study the anti-obesity effect of HT048, composed of *C. pinnatifida* leaf and *C. unshiu* peel, on adipogenesis and lipogenesis in liver and WAT. After 12 weeks of experiment, the body weight and visceral (epididymal, mesenteric and perirenal) fat mass of the animals fed with a diet supplemented with HT048 were significantly lower in HFD-fed mice without affecting the amount of food intakes. WAT accumulation is caused by abnormal regulation of adipocyte differentiation and lipogenesis and associated with obesity [21]. We observed that HT048 supplementation significantly reduced serum TG and TC levels and increased serum HDL-C level. Excessive body weight gain and WAT accumulation are the causes of obesity-related disorders and dyslipidemia characterized by increased TG and decreased HDL-C concentration [22,23]. WAT is to store TG in periods of energy excess and TG is hydrolyzed and free fatty acids are released into the circulation during energy deprivation into the circulation [24]. This result suggests that HT048 improves the serum lipid levels and inhibits adipose tissue accumulation by increasing excretion of lipids in the feces.

We investigated that the cell viability in 3T3-L1 preadipocytes, and also the hepatic AST and ALT enzymes *in vivo*. Chronic treatment with 0.2% and 0.6% of HT048 in the diet did not have toxicity on the liver in high-fat diet fed animals. Moreover, HT048 100 and 300 mg/kg (0.2% and 0.6% of HT048 in the diet) treatment for 12 weeks did not affect serum AST and ALT in the previous study [13]. In other words, 0.2% and 0.6% of HT048 in the diet did not cause a liver injury. However, this study does not explain all the adverse reactions and toxicity. Therefore, we will investigate further experimental study including other toxicity tests.

Obesity is the result of a complicated process regulated by a number of lipogenic and adipogenic factors [25]. SREBP1c is a major transcription factor involved in hepatic lipogenesis induced by HFD and induces the expression of lipogenic genes such as FAS and SCD [26,27]. Our data showed that HT048 supplementation in HFD fed animal down-regulated the expressions of hepatic SREBP1c and its target gene FAS mRNA. HT048 supplementation suppresses hepatic lipogenesis. On the other hand, it up-regulates the mRNA expressions of hepatic β-oxidation-related genes including PPARα and CPT-1 in HFD fed rats. Hepatic PPARα and CPT-1 gene expressions inhibit fat accumulation by up-regulating fatty acid β-oxidation [28]. Thus, our results suggest that HT048 supplementation induces hepatic β-oxidation of fatty acids. PPARγ is the other important key transcription factor in adipogenesis and lipogenesis. It regulates the expression of a number of genes involved the fatty acid synthesis, β-oxidation and adipogenesis including FAS, aP2 and LPL [29,30,31]. Previous studies reported that PPARγ antagonists were effective in the prevention of obesity by inhibiting the adipogenesis [32,33]. aP2 is a mammalian transcription factor and a carrier protein for fatty acids [34]. LPL regulates the disposition of dietary fatty acids as a gatekeeper for fat storage in WAT [35]. In the present study, HT048 significantly decreased the mRNA levels of transcription factor PPARγ and its target genes in the epididymal adipose tissue of HFD-fed obese mice. Our results suggest that decreased body and WAT weights in rat group fed HFD with HT048 supplementation are associated with the down-regulated expression of genes involved in lipogenesis and adipogenesis and up-regulated expression of genes involved in β-oxidation.

In vitro study, we examined Oil red O staining and TG accumulation to evaluate the lipid contents, instead of measuring the cell morphology. Body fat was divided into mesenteric, epididymal, and perirenal fat according to their locations, and their respective weights were measured *in vivo*. HT048 supplementation significantly reduced the white adipose tissue weight and expression of lipogenesis-related genes in epididymal fat. These results make it possible to expect the result of histological analysis.

In the present study, we investigated that the effects of HT048 on abnormal lipid metabolism induced high-fat diet, so we used high-fat diet fed animal model. However, we didn’t study the effect of HT048 on chow diet taking. Therefore, we think that further the studies are necessary to see the effects of the extract on these animals and compare the results to those on HFD.

## 3. Experimental Section

### 3.1. Preparation of HT048

The dried *C. pinnatifida* leaf (Anhui Jinzhai Qiaokang, Jinzhai, China) and dried *C. unshiu* peel (Dong Kyung Pharm, Seoul, Korea) were purchased. The samples were identified by Hocheol Kim and voucher specimens (#HP570 and #HP013) were deposited at the Department of Herbal Pharmacology, College of Korean Medicine, Kyung Hee University, Seoul, Korea. The dried *C. pinnatifida* leaf and dried *C. unshiu* peel were separately extracted twice with 30% ethanol for 4 h at 90 °C in a reflux apparatus. The extracts were filtered and concentrated under reduced pressure, and samples were lyophilized to yield a dark yellow powder. The yield (%) of individual extracts was 14% and 21.5%, respectively. Then, the two kinds of powder were mixed for preparing HT048 in the proportion of the raw materials.

### 3.2. HPLC Analysis of HT048

HT048 extract powder (2 g) was hydrolyzed in 1 M HCl in water (100 mL). The hydrolysis was performed in triplicate. After refluxing at 100 °C for 1 h, the extract was allowed to cool, made up to 200 mL and sonicated for 5 min. The extract was filtered through a 0.45 µm filter for organic solvents, prior to injection.

The hydrolyzed samples were analyzed by using a Waters HPLC system (Water, Milford, MA, USA) comprising a Waters 1525 pump, a 2707 autosampler, and a 2998 PDA detector. The chromatic separation was achieved at 35 °C on a Waters Sunfire™ C18 column (250 mm × 4.6 mm I.D., 5 µm particle size). A binary solvent system was employed consisting of 0.05% phosphoric acid in water as solvent A and tetrahydrofuran-acetonitrile (20:3, *v*/*v*) as solvent B. The gradient program was 0–10 min with 10%–20% solvent B, 10–15 min with 20%–23% B, 15–30 min with 23% B, 30–35 min with 23%–60% B, 35–40 min with 60%–10% B, 40–45 min with 10% B for vitexin. And, for hesperidin, mobile phases A and B were 0.5% phosphoric acid in water (*v*/*v*) and acetonitrile, respectively. Gradient dilution was as follows: 0–15 min with 5%–30% solvent B, 15–20 min with 30%–80% B, 20–25 min with 80-5% B, 25–30 min with 5% B. The flow rate was 1.0 mL/min, and the injection volume was 10 μL. The eluted components were monitored at 284 nm for hesperidin and 330 nm for vitexin.

### 3.3. Cell Culture and Adipocyte Differentiation

3T3-L1 preadipocytes were maintained in DMEM containing 10% BCS, 100 units/mL penicillin, and 100 µg/mL streptomycin. Two-day post-confluent 3T3-L1 preadipocytes, designated as Day 0, were differentiated with DMEM supplemented with 10% FBS, dexamethasone (1 μM), IBMX (0.5 mM), and insulin (10 μg/mL) for 2 days. The medium was then replaced with DMEM containing 10% FBS and insulin (10 μg/mL) and incubated by an additional 2 days. Then it was replaced with fresh DMEM containing 10% FBS every other day. HT048 was dissolved in phosphate-buffered saline and fixed (PBS), directly diluted in DMEM, and treated with different concentrations of HT048 for the course of differentiation.

### 3.4. Cell Viability Assay

3-(4,5-Dimethylthiazol-2-yl)-2.5-diphenyltetrazolium (MTT) reduction was measured to examine the effect of HT048 on cell viability. 3T3-L1 preadipocytes were seeded 1 × 10^4^ cells/well in DMEM containing 10% BCS and incubated at 37 °C in a 5% CO_2_ incubator for 24 h. Cells were treated with different concentrations of HT048 (0, 50, 100, 200, 400 or 800 μg/mL) for 24 h. After treatment, the MTT solution (5 mg/mL) was added to each well and the cells were incubated for 1 h. The resultant formazan product was dissolved by the addition of 100 μL of DMSO. Absorbance was detected at 540 nm using a microplate reader.

### 3.5. Measurement of TG Accumulation and Glycerol in 3T3-L1

Lipolysis was measured using commercial assay kit (BioVision, Milpitas, CA, USA) according to the manufacturer’s protocol. After the treatment, cells were washed two times with lipolysis wash buffer and then incubated for 1 h with lipolysis assay buffer. Media were then collected and samples (50 μL) were assayed for TG and glycerol in a 96-well plate. Absorption was measured at 570 nm.

### 3.6. Oil Red O Staining

Oil red O (ORO) staining was performed on day 8 of the differentiation course to stain the accumulated lipid droplets in differentiated adipocytes. Cells were washed twice with PBS with 10% formaldehyde for 1 h at room temperature. After the formaldehyde was discarded, the cells were washed with PBS and distilled water three times. They were stained with ORO dye/60% isopropanol solution for 10 min and the absorbance was measured by a microplate reader.

### 3.7. Animal Experiments

Three weeks old Sprague-Dawley male rats were obtained from Samtako (Gyeonggi-do, Korea). The experimental procedures were performed in accordance with the animal care guidelines of the Korea Institute of Science and Technology for Eastern Medicine (KISTEM)’s institutional animal care and use committee (protocol no. KISTEM-IACUC-2014-003). Animals were housed in polycarbonate cages and maintained at 23 ± 1 °C, 55%–60% relative humidity, and a 12 h light/dark cycle. Rats were fed a normal chow diet for acclimation for 1 week after delivery. At 4 week of age, animals were then randomly divided into five groups (*n* = 12) and fed the chow or experimental diets for 12 weeks: chow control, 60% high-fat diet (HFD) control (D12492, Research Diets Inc.), HFD combined with 0.04% orlistat, HFD combined with 0.2% HT048, and HFD combined with 0.6% HT048. They were provided free access to food and distilled water every 2–3 days. At the end of the experimental period, rats were sacrificed after 12 h of fasting. Blood samples were collected and centrifuged 1000× *g* for 15 min at 4 °C, and the serum was separated to analyze the plasma biomarkers. After blood collection, the liver and adipose tissues were promptly removed, rinsed, weighed and stored at −70 °C.

### 3.8. Serum Measurements

Serum triglyceride (TG), total cholesterol (TC), alanine transaminase (ALT), and aspartate aminotransferase (AST) concentrations were measured by VetTest 8008 (IDEXX Lab Inc., Westbrook, ME, USA). Serum high-density lipoprotein cholesterol (HDL-C) was determined using an ELISA kit (BioVision).

### 3.9. Analysis of Fecal Lipids

Feces were obtained during the last 7 days. Fecal samples (1 g) were ground to a fine powder. Total fecal lipid extractions were measured as described by method of Folch *et al.* [36]. Briefly, fecal samples were extracted with chloroform–methanol (2:1, *v*/*v*, 4 mL) in a screw-capped glass tube. The extract was dried and weighed. The amounts of neutral fat excreted in the feces were estimated as the averages in mg/g.

### 3.10. Real-Time Quantitative PCR Analysis

Total RNAs from mouse liver and adipose tissue were extracted using Trizol reagent (Invitrogen, Carlsbad, CA, USA) according to the manufacturer’s instruction. Total RNAs (200 ng) were reverse-transcribed using High capacity cDNA reverse transcription kit (Applied Biosystems, Foster, CA, USA) according to the manufacturer’s instruction. Real-time quantitative polymerase chain reaction (PCR) was performed on a 7300 Fast Real Time PCR system (Applied Biosystems) using Power SYBR Green PCR Master Mix (Applied Biosystems). Primers were designed using nucleotide sequence and synthesized by Bioneer (Daejeon, Korea). The following primers were used: SREBP1c, forward 5′-CGCTACCGTTCCTCTATCA-3′ and reverse 5′-TCGCAGGGTCAGGTTCT-3′; FAS, forward 5′-GGATGTCAACAAGCCCAAGT-3′ and reverse 5′-AACTGCAGCAACTTTAATATACGCTATT-3′; Peroxisome proliferator-activated receptor alpha (PPARα), forward 5′-TGGAGTCCACGCATGTGAAG-3′ and reverse 5′-CGCCAGCTTTAGCCGAATAG-3′; Carnitine palmitoyltransferase 1 (CPT-1), forward 5′-TAGGACAGGCAGAAAATTGC-3′ and reverse 5′-CAGTAGGAGCCGATTCAAAA-3′; PPARγ, forward 5′-CCCTGGCAAAGCATTT GTAT-3′ and reverse 5′-GGTGATTTGTCTGTTGTCTTTCC-3′; aP2, forward 5′-GGCTTCGCCACCAGGAA-3′ and reverse 5′-CCCTTCTACGCTGATGATCAAGT-3′; LPL, forward 5′-CAGCAAGGCATACAGGTG-3′ and reverse 5′-CGAGTCTTCAGGTACATCTTAC-3′ CEBP/α, forward 5′-TGGCCGGCCTCTTCCC-3′ and reverse 5′-GGCTGCAGGTGCATGGTGGT-3′; The cycling conditions were as follows: 15 min at 95 °C, 40 cycles of 15 s each at 94 °C, and 30 s at 72 °C. Relative quantification was calculated using the Delta-Delta method [37].

### 3.11. Statistical Analysis

Statistical analysis was performed by using the SAS package (release 9.3, SAS Institute Inc., Cary, NC, USA). Data are expressed as the means ± standard deviation (S.D.). One-way ANOVA and the Duncan’ multiple test were used to determine the statistical differences between the treatment groups. A value of *p* < 0.05 was considered statistically significant.

## 4. Conclusions

In conclusion, HT048, a combination of *C. pinnatifida* leaf and *C. unshiu* peel extracts, inhibits adipocyte differentiation by down-regulating the expression of adipogenesis-related genes in 3T3-L1 preadipocytes. It reduces body weight and fat accumulation by modulating the expression of lipid metabolism-related genes in HFD-induced obese rats. These results suggest that HT048 may be a potential candidate for the control of obesity, hyperlipidemia and its related metabolic disorders.

## References

[1] Bray G.A., Bellanger T. (2006). Epidemiology, trends, and morbidities of obesity and the metabolic syndrome. Endocrine.

[2] Gurevich-Panigrahi T., Panigrahi S., Wiechec E., Los M. (2009). Obesity: Pathophysiology and clinical management. Curr. Med. Chem..

[3] Haslam D.W., James W.P. (2005). Obesity. Lancet.

[4] Ahmed S.M., Clasen M.E., Donnelly J.E. (1998). Management of dyslipidemia in adults. Am. Fam. Physician.

[5] Shimano H., Yahagi N., Amemiya-Kudo M., Hasty A.H., Osuga J., Tamura Y., Shionoiri F., Iizuka Y., Ohashi K., Harada K. (1999). Sterol regulatory element-binding protein-1 as a key transcription factor for nutritional induction of lipogenic enzyme genes. J. Biol. Chem..

[6] Rosen E.D., Spiegelman B.M. (2000). Molecular regulation of adipogenesis. Ann. Rev. Cell. Dev. Biol..

[7] Heck A.M., Yanovski J.A., Calis K.A. (2000). Orlistat, a new lipase inhibitor for the management of obesity. Pharmacotherapy.

[8] Bray G.A. (2001). Drug treatment of obesity. Rev. Endocr. Metab. Disord..

[9] Chang Q., Zuo Z., Harrison F., Chow M.S. (2002). Hawthorn. J. Clin. Pharmacol..

[10] Kuo D.H., Yeh C.H., Shieh P.C., Cheng K.C., Chen F.A., Cheng J.T. (2009). Effect of shanzha, a chinese herbal product, on obesity and dyslipidemia in hamsters receiving high-fat diet. J. Ethnopharmacol..

[11] Assini J.M., Mulvihill E.E., Huff M.W. (2013). Citrus flavonoids and lipid metabolism. Curr. Opin. Lipidol..

[12] Lee Y.S., Cha B.Y., Choi S.S., Choi B.K., Yonezawa T., Teruya T., Nagai K., Woo J.T. (2013). Nobiletin improves obesity and insulin resistance in high-fat diet-induced obese mice. J. Nutr. Biochem..

[13] Lim D., Song M., Park J., Park S., Kim N., Gaire B., Choi H.-Y., Kim H. (2012). Anti-obesity effect of HT048, a herbal combination, in high fat diet-induced obese rats. Molecules.

[14] Woods S.C., Seeley R.J., Rushing P.A., D’Alessio D., Tso P. (2003). A controlled high-fat diet induces an obese syndrome in rats. J. Nutr..

[15] Ali A.T., Hochfeld W.E., Myburgh R., Pepper M.S. (2013). Adipocyte and adipogenesis. Eur. J. Cell. Biol..

[16] Yang J.-W., Kim S. (2015). Ginsenoside rc promotes anti-adipogenic activity on 3T3-L1 adipocytes by down-regulating C/EBPα and PPARγ. Molecules.

[17] Kim J., Jang D., Kim H., Kim J. (2009). Anti-lipase and lipolytic activities of ursolic acid isolated from the roots of actinidia arguta. Arch. Pharm. Res..

[18] Tang Q.Q., Otto T.C., Lane M.D. (2003). Mitotic clonal expansion: A synchronous process required for adipogenesis. Proc. Natl. Acad. Sci. U.S.A..

[19] Fève B. (2005). Adipogenesis: Cellular and molecular aspects. Best Pract. Res. Clin. Endocrinol. Metab..

[20] Rosen E.D., MacDougald O.A. (2006). Adipocyte differentiation from the inside out. Nat. Rev. Mol. Cell. Biol..

[21] Moller D.E., Flier J.S. (1991). Insulin resistance—Mechanisms, syndromes, and implications. New Engl. J. Med..

[22] Paccaud F., Schlüter-Fasmeyer V., Wietlisbach V., Bovet P. (2000). Dyslipidemia and abdominal obesity: An assessment in three general populations. J. Clin. Epidemiol..

[23] Modan M., Halkin H., Almog S., Lusky A., Eshkol A., Shefi M., Shitrit A., Fuchs Z. (1985). Hyperinsulinemia. A link between hypertension obesity and glucose intolerance. J. Clin Invest..

[24] Schweiger M., Schreiber R., Haemmerle G., Lass A., Fledelius C., Jacobsen P., Tornqvist H., Zechner R., Zimmermann R. (2006). Adipose triglyceride lipase and hormone-sensitive lipase are the major enzymes in adipose tissue triacylglycerol catabolism. J. Biol. Chem..

[25] Zhang X.H., Huang B., Choi S.K., Seo J.S. (2012). Anti-obesity effect of resveratrol-amplified grape skin extracts on 3T3-L1 adipocytes differentiation. Nutr. Res. Pract..

[26] Horton J.D., Goldstein J.L., Brown M.S. (2002). Srebps: Activators of the complete program of cholesterol and fatty acid synthesis in the liver. J. Clin. Invest..

[27] Kim J.B., Spiegelman B.M. (1996). ADD1/SREBP1 promotes adipocyte differentiation and gene expression linked to fatty acid metabolism. Genes Dev..

[28] Chawla A. (2010). Control of macrophage activation and function by ppars. Circ. Res..

[29] Gregoire F.M., Smas C.M., Sul H.S. (1998). Understanding adipocyte differentiation. Physiol. Rev..

[30] Schoonjans K., Staels B., Auwerx J. (1996). The peroxisome proliferator activated receptors (PPARs) and their effects on lipid metabolism and adipocyte differentiation. Biochim. Biophys. Acta.

[31] Son Y., Nam J.S., Jang M.K., Jung I.A., Cho S.I., Jung M.H. (2013). Antiobesity activity of vigna nakashimae extract in high-fat diet-induced obesity. Biosci. Biotechnol. Biochem..

[32] Zhang Y., Fan S., Hu N., Gu M., Chu C., Li Y., Lu X., Huang C. (2012). Rhein reduces fat weight in db/db mouse and prevents diet-induced obesity in c57bl/6 mouse through the inhibition of ppargamma signaling. PPAR Res..

[33] Zhang Y., Yu L., Cai W., Fan S., Feng L., Ji G., Huang C. (2014). Protopanaxatriol, a novel ppargamma antagonist from panax ginseng, alleviates steatosis in mice. Sci. Rep..

[34] Foster D.W. (2004). The role of the carnitine system in human metabolism. Ann. N.Y. Acad. Sci..

[35] Fielding B.A., Frayn K.N. (1998). Lipoprotein lipase and the disposition of dietary fatty acids. Br. J. Nutr..

[36] Folch J., Lees M., Sloane Stanley G.H. (1957). A simple method for the isolation and purification of total lipides from animal tissues. J. Biol. Chem..

[37] Schmittgen T.D., Livak K.J. (2008). Analyzing real-time pcr data by the comparative C(T) method. Nat. Protoc..

